# Supplementary material for: Persisting volcanic ash particles impact stratospheric SO2 lifetime and aerosol optical properties
Source: Nat Commun. 2020 Sep 10;11:4526. doi: 10.1038/s41467-020-18352-5 (PMC7483524; doi:10.1038/s41467-020-18352-5)
Supplement: Supplementary file 1 — Supplementary Information [file 41467_2020_18352_MOESM1_ESM.pdf]

Persisting volcanic ash particles impact stratospheric SO_2 lifetime and aerosol optical properties

Zhu et al.

Supplementary Notes

Supplementary Note 1. Applying OMI observational bias in the model output

It is difficult to consider the observational noise in the model output. The detection limit for OMI is 5 adjacent pixels with SO_2 columns larger than 5 times the noise level[1]. A model grid cell is ~ 150 times larger than one OMI pixel. SO_2 likely partially occupies the model grid cells with higher SO_2 concentration than the gridbox average. Therefore, we cannot simply pick the detection limit to eliminate SO_2 in the simulation. The green shaded area in Fig. 2 shows two extreme estimations of SO_2 burden in our best model case (SO2onAsh) considering the observational uncertainty. The lower boundary of the shaded area only counts the grid cell with the gridbox average SO_2 higher than the detection limit. To create the upper boundary of the shaded area, we assume the simulated SO_2 occupies only a part of the model grid cell equivalent to the 5 adjacent pixel threshold of OMI and compare that with the OMI detection limit. During the first couple of days, the uncertainty is small (the green shaded area is narrow). Except for the SO2onash case, all the other model cases cannot reduce the SO_2 to be within the green shaded area, which indicates the need to consider the reaction of SO_2 on ash. The uncertainties increase with time. The green solid line is in the middle of the green shaded area and represents, on average, the SO_2 occupying 20% of the gridbox. Due to the mismatch between OMI pixel sizes and our grid sizes, we pick the noise level instead of the detection limit to apply in the model output in order to compare with the satellite observations.

Supplementary Note 2. SO_2 and ash horizontal distribution

We explore the SO_2 and ash horizontal distributions after the eruption and make comparisons between simulations and OMI and CALIPSO data. The model is nudged with the Modern-Era Retrospective Analysis for Research and Applications (MERRA) for temperature, zonal and meridional winds, and surface pressure fields. We find the simulated winds show an abrupt change of direction with latitude near the injection site, but little vertical wind shear. Due to limited spatial resolution the simulated wind field may not reproduce the actual wind shear and meridional wind speed accurately. In reality there must be vertical wind shear to reproduce the path taken by the volcanic material. Supplementary Fig. 1a shows the radiosonde wind observation from the Integrated Global Radiosonde Archive at Juanda Surabaya, a station 83 km northeast of Mt. Kelut. It shows the simulated winds capture the general trend in the observations, but not the large variation in direction and speed. Supplementary Fig. 1b shows the simulated zonal and meridional winds at 61 hPa (~19.5 km) on Feb. 14th. Mt. Kelut is located right where the wind direction changes from east to west. Also, the simulated meridional wind is usually less than 4 m s^{-1} near Mt. Kelut (blue star), but observed winds can be higher or lower.

The spreading of the volcanic aerosol layer as a function of time provides clues to the vertical and horizontal distributions of the ash injection and the winds. According to the CALIPSO backscatter observation, the ash was transported both west and east, and slightly north and south. After 20 days, CALIPSO observed the volcanic aerosol layer to the east of the volcano near the coast of Peru (0˚S, 80˚W) and to the west of the volcano near Brasília, Brazil (15˚S, 45˚W).

In order to match the CALIPSO spreading pattern in Supplementary Fig. 2, we conduct several simulations by injecting the ash into different latitudes and compare the locations of ash layers with CALIPSO observations. We find that injecting the ash in a latitude band from 2.8˚ S to 10˚ S (the red box in Supplementary Fig. 1) can generally reproduce the CALIPSO observed volcanic aerosol layer distribution. On February 19th (6 days after the eruption), the simulations and the observations show the volcanic aerosols move both west and east. On March 4th (19 days after the eruption), the volcanic aerosol layer is transported east near the east coast of Peru and transported west near the west coast of Brazil.

We compare the SO_2 distribution from several simulated cases (described in Table 1) and the OMI data (Supplementary Fig. 3). We inject SO_2 into a 10-degree latitude band for the Base case and the SO2onAsh case. Both cases show similar transport patterns, but the Base case has higher concentration values. On Feb. 20th, OMI shows a peak concentration of 1.5 DU near 50˚E, while the Base case value is near 1.2 DU and the SO2onAsh is near 0.6 DU. The Reference case shows a similar concentration on the first day, but with a narrower longitudinal range and peaking near 100˚E on Feb. 20th. Neither of these cases can explain the detailed distribution shown by OMI.

Due to the uncertainty of the SO_2 spreading pattern, we test simulations with and without injecting SO_2 into a 10-degree latitude band to determine if the injection pattern impacts the SO_2 lifetime. We find the SO_2 injection pattern does not affect our conclusion that the uptake of SO_2 on ash is the major control for SO_2 lifetime. We need to tune the saturation coverage or uptake efficiency for each simulation, but the tuning is still within the uncertainties of these parameters. Supplementary Fig. 11 shows two model cases when we inject both SO_2 and ash into one grid box. Supplementary Fig. 11 shows that we need to tune the uptake efficiency from $3x10^{-3}$ to $8x10^{-4}$ to match the observation. Note that using a uptake efficiency of $8x10^{-4}$ and a saturation coverage of $3x10^{16}$ molecules cm^{-2} is not the only way to match the data we discuss in Supplementary Note 3.

We suggest using a finer resolution model in the first couple of days to help with the chemical component and particle transport patterns in the climate model.

Supplementary Note 3. The SO_2 reaction on ash and mineral dust references

Laboratory data for the uptake efficiency of SO_2 on Mt. Kelut eruption ash are not available. However, there are several previous investigations of SO_2 uptake by mineral aerosol and volcanic glass and ash from other eruptions. Mineral dust and volcanic ash have several similarities, as well as differences[2]. For example, the main chemical elements in both are silicon and oxygen as they originate from the Earth's crust and mantle. Further, both have complex morphologies and specific surfaces areas that are often 100 times larger than the geometric surface area of spheres[3,4]. For these reasons, insight into the reactivity of ash might be gained by examining past literature on mineral aerosol particles.

Usher et al.[4] used a low-pressure Knudsen cell to determine the initial uptake efficiency of SO_2 on several mineral samples, a China Loess dust sample, and a pure SiO_2 surface. While no SO_2 uptake was observed on pure SiO_2, on all other samples they found a large initial uptake that began to saturate over time. After correcting for the BET surface area of their dust samples, they reported an initial uptake efficiency in the range 7.0×10^{-5} to 5.1×10^{-4} for the mineral samples and 3×10^{-5} for the China Loess sample. Geometric surface area is the smallest possible area for a given mass. The BET surface area is derived from gas adsorption, such as multilayer absorption, and includes interior surfaces such as inside pores. They proposed that the uptake occurs by SO_2 reacting with the surface oxygen or hydroxyl groups to form SO_3^{2-} or HSO_3^-, respectively. Upon exposure of the adsorbed S to ozone, they further detected oxidation of S (IV) to S (VI). Similarly, Huang et al.[5] found initial uptake efficiencies of SO_2 on dry Arizona test dust, Tengger desert dust, and Arizona test dust (ATD) to be in the range 0.35×10^{-4} to 2.29×10^{-4} after correcting for limited gas phase diffusion into the sample pores. Other studies have reported lower initial uptake efficiencies in the 10^{-6} range[6,7]. The differences may be attributed to differences in assumed sample surface area, porosity, and chemical composition. Finally, increases in relative humidity[5] and UV radiation[7] can significantly influence the SO_2 uptake and oxidation to sulfate.

While the literature on SO_2 uptake by volcanic ash samples is much more limited, there are two recent laboratory studies providing uptake data. Maters et al.[8] used a Knudsen cell to measure the initial uptake efficiency of SO_2 on eight different samples of volcanic glass and ash. The composition spanned a range of compositions from trachybasaltic to rhyolitic to cover the range of material produced in different types of volcanic eruptions. The time dependence of the SO_2 uptake observed on volcanic materials was very similar to that observed on mineral aerosol, with

a large initial uptake followed by a much slower uptake and saturation over time. While all of the samples had similar reactivity, the dacite sample is perhaps the most representative of the Kelut eruption[9]. For a temperature of ~250 K, the initial uptake efficiency was found to be 1.3×10^{-2} for dacite glass[8]. The initial uptake efficiencies in Maters et al.[8] were calculated assuming a geometric surface area for their sample holder. Urupina et al.[10] measured the steady-state uptake efficiency for SO_2 on natural volcanic dusts. While they showed an SO_2 uptake curve that exhibited strong initial uptake followed by saturation, they only reported the uptake efficiency at long times after 10 hr of exposure to obtain a steady-state uptake. At steady state, they found low uptake efficiencies in the range 10^{-9} to 10^{-8}. This low uptake result is not surprising as the sample may have been nearly saturated at this time. Because of the differing timescales, it is not possible to directly compare the uptake kinetics between Maters et al.[8] and Urupina et al.[10]. However, both studies do report the total number of SO_2 molecules taken up by the volcanic samples over the course of the experiment normalized to the BET surfaces areas. For dacite, the saturation coverage was found to be 3.1×10^{13} molecules cm^{-2} assuming the BET surface area[8]. Similarly, Urupina et al.[10] report coverages of 0.96 to 2.5×10^{13} molecules cm^{-2} for their 5 volcanic samples, again assuming the BET surface area.

Supplementary Fig. 5 illustrates simulations of SO_2 with various choices of uptake efficiency γ and saturation coverage. It shows that larger γ values or larger saturation coverages result in greater losses of SO_2 in the first couple of days. Also, several different combinations of γ and saturation coverage can explain the observed SO_2 burden (red lines). As we increase the γ, we can balance the results by decreasing the saturation coverage. The saturation coverage in Supplementary Fig. 5a is towards the upper limit of saturation coverage. γ in Supplementary Fig. 5c is too high based on laboratory data. Also, if the saturation coverage is too low (Supplementary Fig. 5d), we cannot compare it well with the observation even with a very high γ. The test cases in Supplementary Fig. 5 suggest the reasonable saturation coverage is in the 10^{16} molecules cm^{-2} range, and γ is in the 10^{-3} to 10^{-2} range assuming geometric surface area.

Supplementary Figures

Supplementary Fig. 1: The wind speed profile and wind field after Mt. Kelut eruption

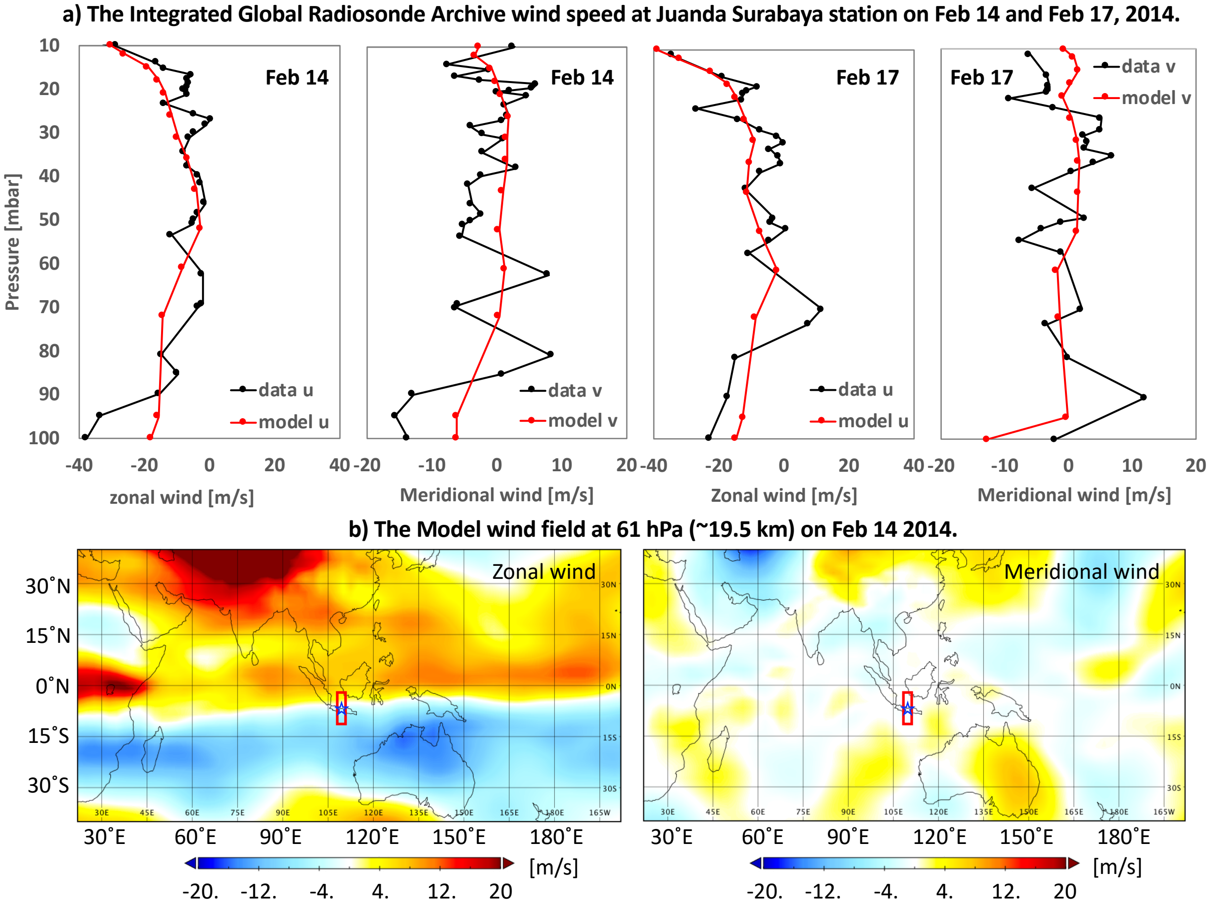

Panel a: Radiosonde wind data (black) at Juanda Surabaya (7.4˚S, 112.8˚E) compared with model nudged wind (red); Panel b: The simulated wind field at 61 hPa (~19.5 km). The blue stars mark the location of Mt. Kelut volcano. The red boxes are the latitude bands for the ash and SO_2 injection in the Base case.

Supplementary Fig. 2: The total backscatter distribution of the sulfate/ash layer

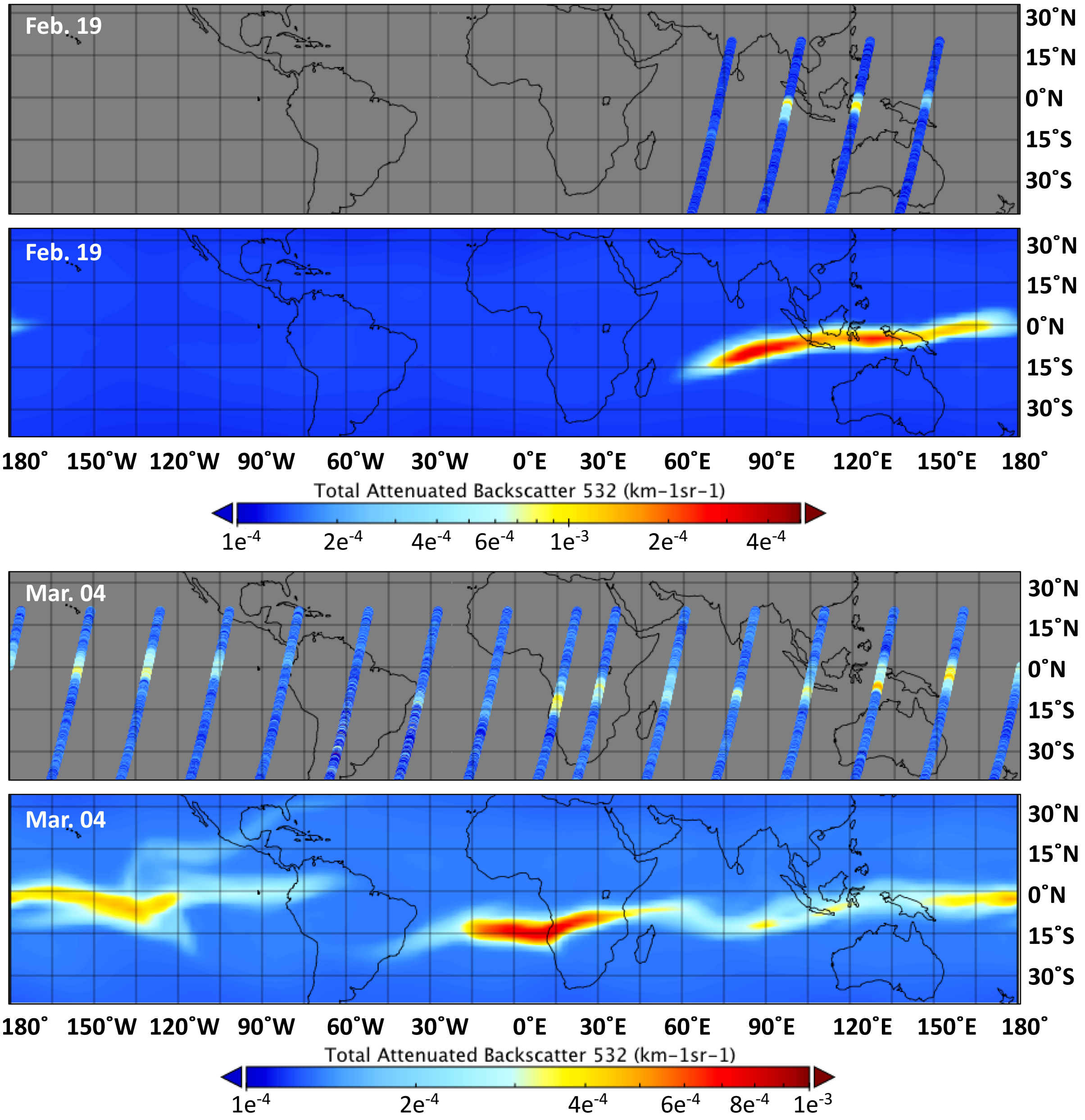

The distribution of the sulfate/ash layer observed by CALIPSO (colored stripes) and from the simulation on Feb. 19th and March 4th, 2014. The data shows the total backscatter averaged from 72-52 hPa for the simulation and from 18.5-20.5 km for the CALIPSO observation.

Supplementary Fig. 3: The SO_2 column evolution

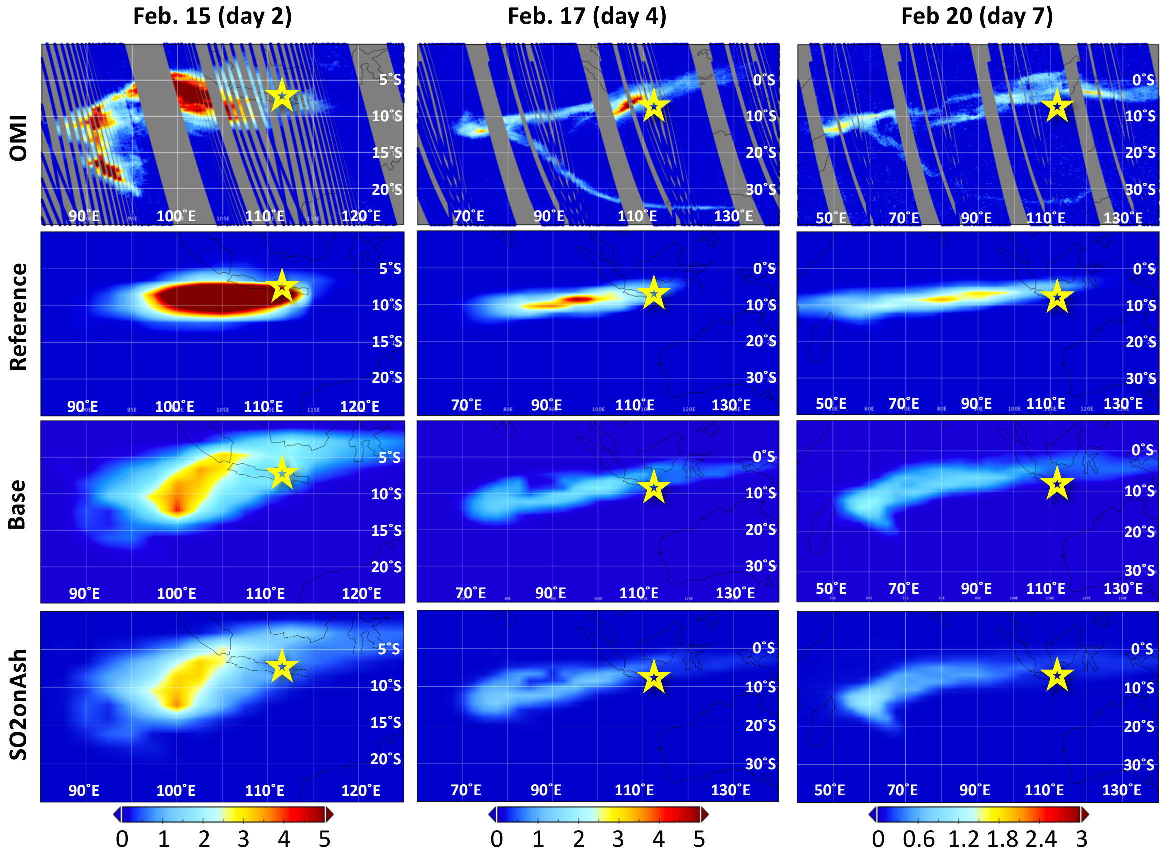

The SO_2 column evolution from OMI and three model cases, representing the Reference case, the Base case and the SO2onAsh case (detailed in Table 1). The yellow stars indicate the location of Mt. Kelut. The color bar is in Dobson units.

Supplementary Fig. 4: OH concentration at 61 hPa

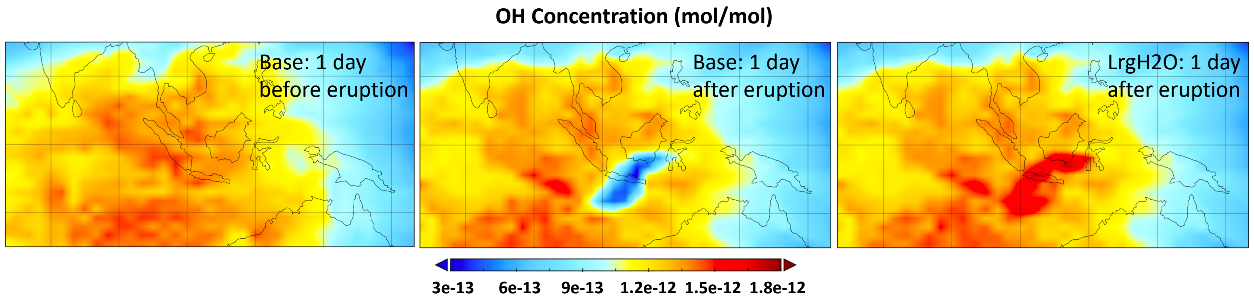

OH concentration at 61 hPa one day before and after the eruption from the Base case and the LrgH2O case.

Supplementary Fig. 5: Test cases with various uptake efficiency and surface saturation coverage

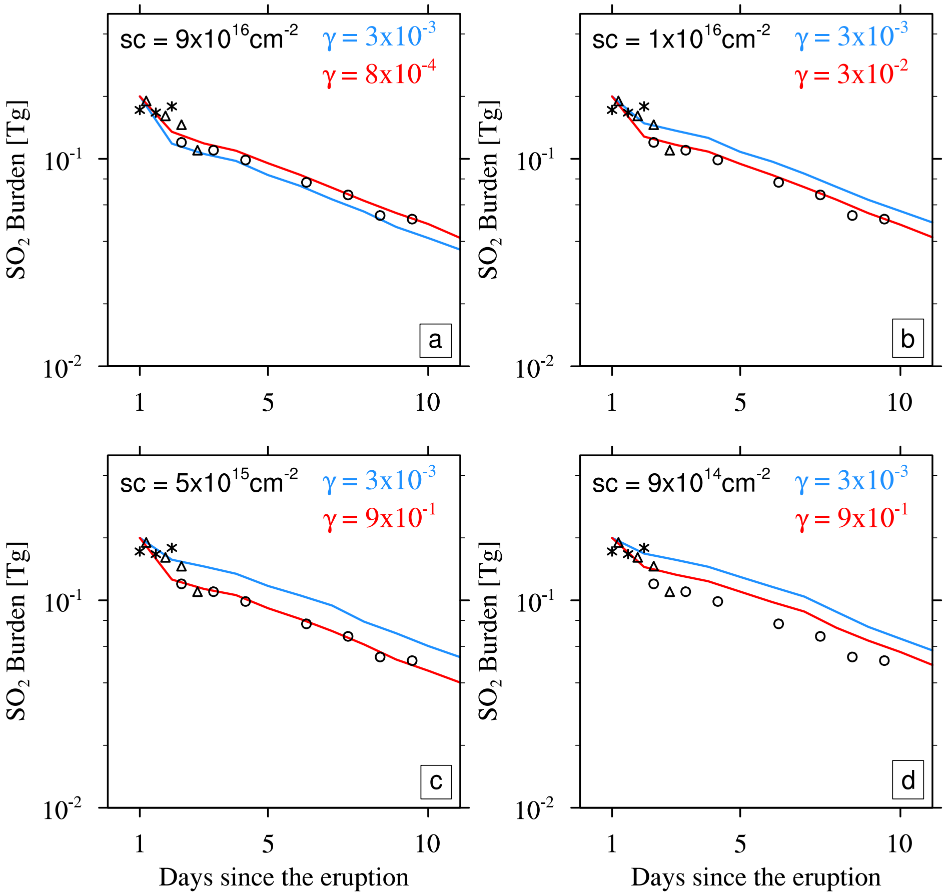

γ is the uptake efficiency and sc is the surface saturation coverage. The symbols are the same observations as Fig. 2.

Supplementary Fig. 6: The falling distance for different particle shapes and densities

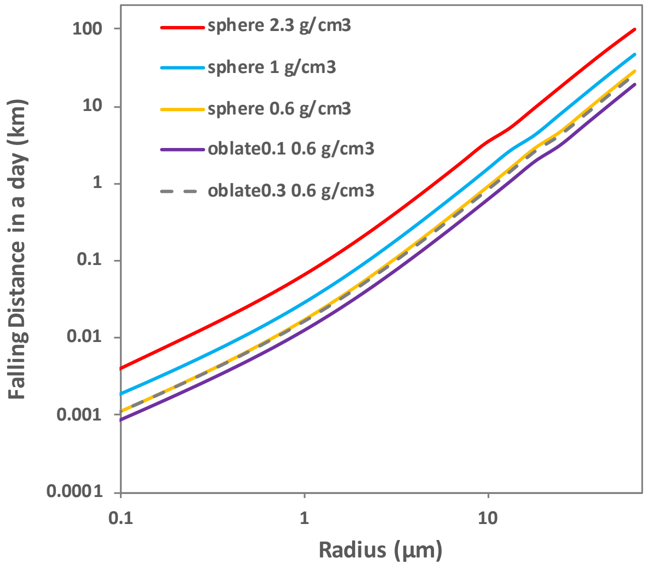

The purple line is the case assuming particles with length/diameter ratios of 0.1. The grey dash line is the case assuming particles with length/diameter ratios of 0.3.

Supplementary Fig. 7: The particle number size distributions

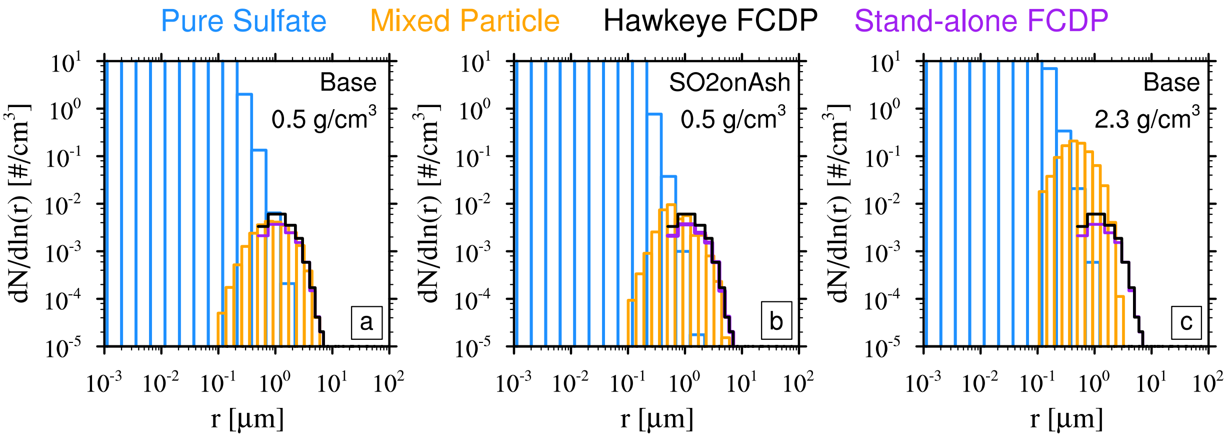

The orders of the figures are the same as in Fig. 4.

Supplementary Fig. 8: The un-normalized mixed particle size distribution at 61 hPa

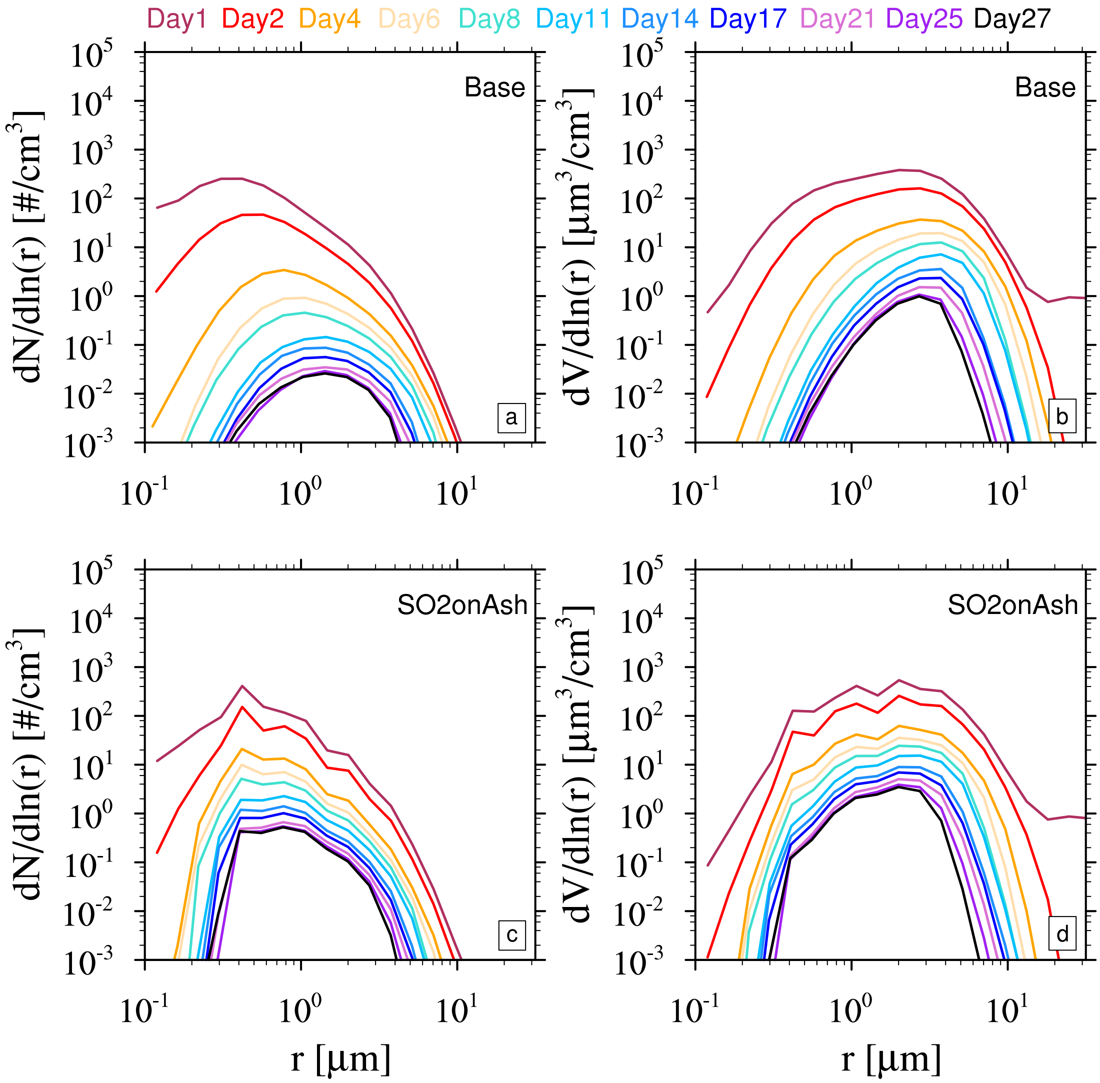

The un-normalized mixed particle size distribution at 61 hPa (19.5 km) for the Base case and the SO2onAsh case. The values are picked at the location with the largest mass mixing ratio of mixed particles on the days specified on top of the figure.

Supplementary Fig. 9: The un-normalized pure sulfate size distribution at 61 hPa

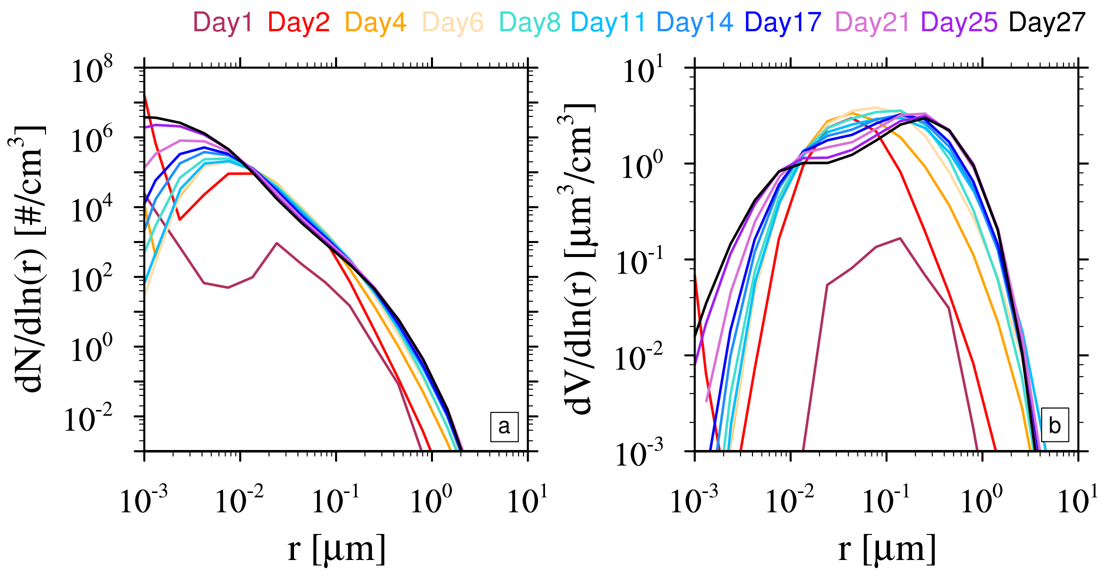

The un-normalized pure sulfate size distribution at 61 hPa (19.5 km) for the SO2onAsh case. The values are picked at the location with the largest mass mixing ratio of pure sulfate particles on the days specified on top of the figure.

Supplementary Fig. 10: The composition evolution from the Base case.

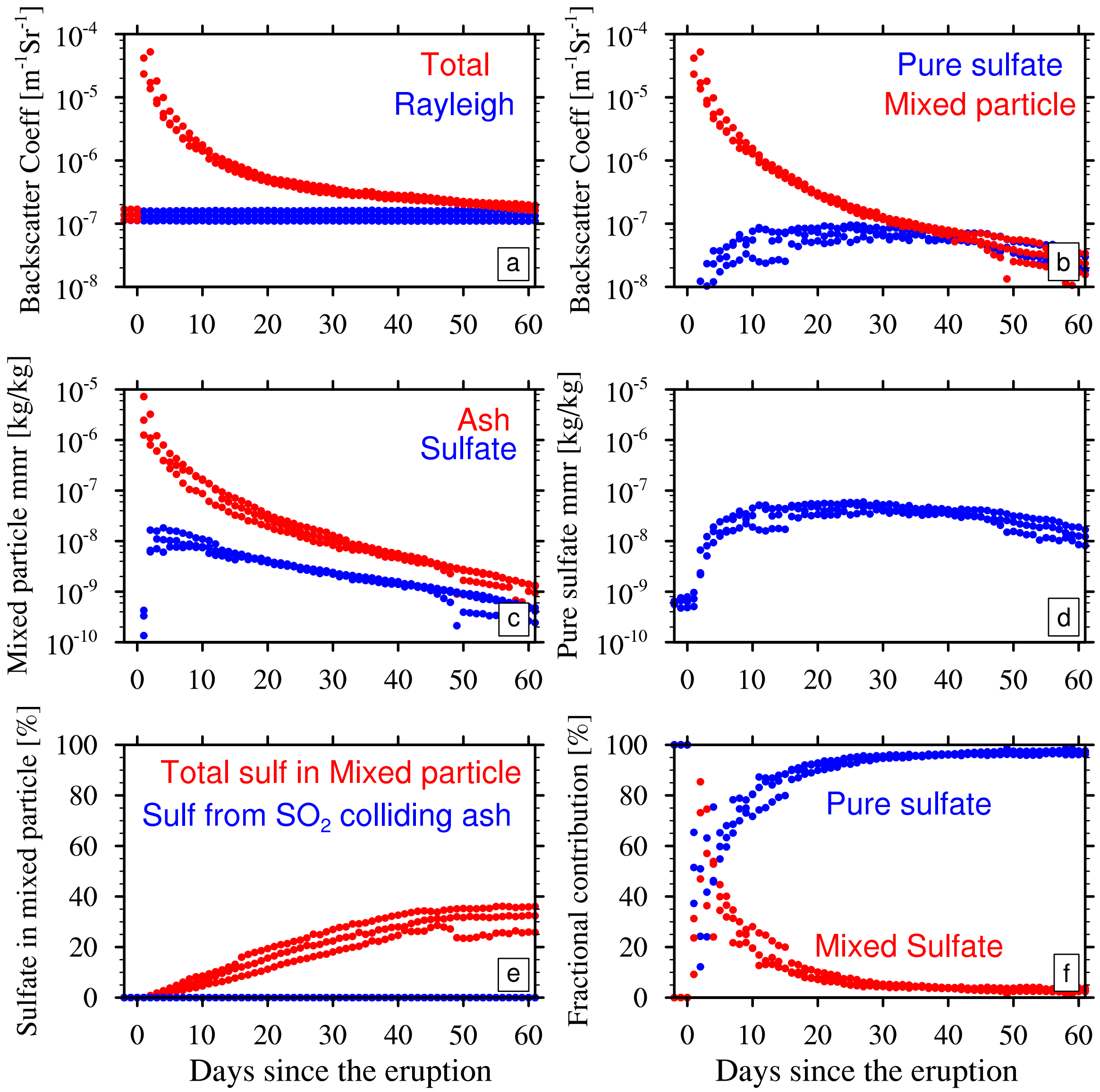

The orders of the figures are the same as in Fig. 7.

Supplementary Fig. 11: The SO_2 evolution when we inject both SO_2 and ash into one grid box

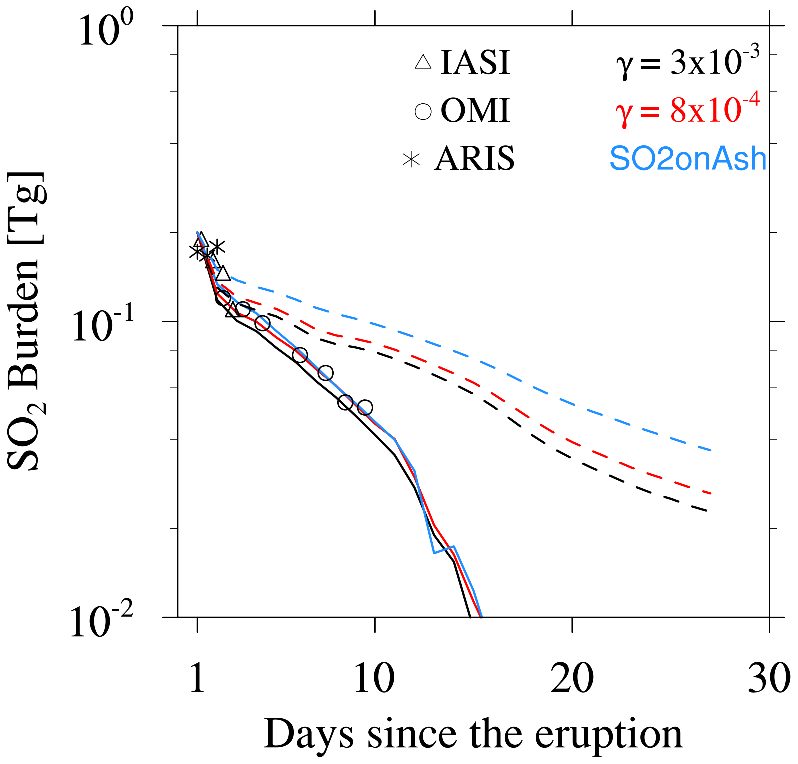

The volcanic SO_2 burden from satellite observations (symbols) and two model simulations (lines). The definitions of dashed lines and solid lines are the same as Fig. 2. The black lines use the uptake efficiency of $3x10^{-3}$ and the saturation coverage of $3x10^{16}$ molecules cm^{-2}; The red lines use the uptake efficiency of $8x10^{-4}$ and the saturation coverage of $3x10^{16}$ molecules cm^{-2}. The blue lines are the SO2onAsh case shown in Fig. 2.

Supplementary Reference:

1. Krotkov, N. A., Schoeberl, M. R., Morris, G. A., Carn, S. & Yang, K. Dispersion and lifetime of the SO2 cloud from the August 2008 Kasatochi eruption. *Journal of Geophysical Research: Atmospheres* **115**, (2010).
2. Langmann, B. Volcanic ash versus mineral dust: atmospheric processing and environmental and climate impacts. *ISRN Atmospheric Sciences* **2013**, (2013).
3. Riley, C. M., Rose, W. I. & Bluth, G. J. S. Quantitative shape measurements of distal volcanic ash. *Journal of Geophysical Research: Solid Earth* **108**, (2003).
4. Usher, C. R., Al-Hosney, H., Carlos-Cuellar, S. & Grassian, V. H. A laboratory study of the heterogeneous uptake and oxidation of sulfur dioxide on mineral dust particles. *Journal of Geophysical Research: Atmospheres* **107**, ACH 16-1 (2002).
5. Huang, L., Zhao, Y., Li, H. & Chen, Z. Kinetics of heterogeneous reaction of sulfur dioxide on authentic mineral dust: effects of relative humidity and hydrogen peroxide. *Environmental science & technology* **49**, 10797–10805 (2015).
6. Ullerstam, M., Johnson, M. S., Vogt, R. & Ljungström, E. DRIFTS and Knudsen cell study of the heterogeneous reactivity of SO 2 and NO 2 on mineral dust. *Atmospheric Chemistry and Physics* **3**, 2043–2051 (2003).
7. Park, J. & Jang, M. Heterogeneous photooxidation of sulfur dioxide in the presence of airborne mineral dust particles. *Rsc Advances* **6**, 58617–58627 (2016).
8. Maters, E. C., Delmelle, P., Rossi, M. J. & Ayris, P. M. Reactive uptake of sulfur dioxide and ozone on volcanic glass and ash at ambient temperature. *Journal of Geophysical Research: Atmospheres* **122**, 10–077 (2017).

9. Cassidy, M. *et al.* Explosive eruptions with little warning: experimental petrology and volcano monitoring observations from the 2014 eruption of Kelud, Indonesia. *Geochemistry, Geophysics, Geosystems* **20**, 4218–4247 (2019).

10. Urupina, D. *et al.* Uptake and surface chemistry of SO2 on natural volcanic dusts. *Atmospheric Environment* **217**, 116942 (2019).
